# It’s not difficulty that matters, but strategy: Perceived stressor, functional and dysfunctional coping strategies in ultra-trails of extreme duration

**DOI:** 10.1371/journal.pone.0332058

**Published:** 2025-09-12

**Authors:** Pietro Trabucchi, Barbara Pellegrini, Aldo Savoldelli, Gianandrea Giacoma, Ilaria Vergine, Carlo Galimberti, Sara Garofalo, Federico Schena

**Affiliations:** 1 CERISM, Centro Ricerca Interuniversitario Sport Montagna e Salute, Rovereto, Italy; 2 Department of Engineering for Innovation in Medicine, University of Verona, Verona, Italy; 3 CIBIO, Department of Cellular, Computational and Integrative Biology; University of Trento, Trento, Italy; 4 Research Center in Communication Psychology (PsiCom), Università Cattolica, Milan, Italy; 5 Independent researcher, Milano, Italy,; 6 Department of Psychology, University of Bologna, Bologna, Italy; 7 Department of Neuroscience, Biomedicine and Movement, University of Verona, Verona, Italy; University of Mississippi, UNITED STATES OF AMERICA

## Abstract

To date, no studies have proposed specific taxonomies of stressors and coping strategies used to manage them by ultra trail runners in races longer than 200 miles, with existing research focusing on significantly shorter distances an then on challenges that could be of a different nature. The aim of this study was to fill this gap by developing specific taxonomies that would group both the stressors encountered and the coping strategies into distinct conceptual categories. Furthermore, to observe in a real competition if these taxonomies allows the evaluation of the coping strategies used by athletes. Two Focus Groups composed of experts on the topic proposed provisional classifications, which were analysed using Applied Thematic Analysis. Three distinct Draft Taxonomies were identified: typical stressors, functional and dysfunctional coping strategies used by athletes during competitions. A critical comparison between the provisional taxonomies and literature led to the development of three definitive taxonomies. In a second phase, to verify whether the taxonomies reflected the real experiences of runners, seven participants in a 280-mile race were interviewed daily during the event. Their responses regarding the stressors and the strategies they had implemented to deal with them were recorded. Expert panelists categorized the transcribed responses according to the proposed taxonomies. The concordance of the judgments, verified with the Fleiss K, was considered a measure of the taxonomies’ ability to capture real experiences. Results confirmed substantial agreement between the raters regarding both the stressors (K = 0.711, p < 0.001) and the coping strategies (K = 0.73, p < 0.001). The analysis of the proportion between the use of functional and dysfunctional strategies proved to be high (between 0.66 and 1) among athletes who completed more than 50% of the race. The taxonomies were found to effectively described athletes’ race experiences, revealing context-specific coping strategies likely developed through years of practice.

## Introduction

Ultra trail represents a sporting discipline that is increasingly known and practiced worldwide [1]. In general, according to the International Trail Running Association, a trial race is “a pedestrian competition open to everyone, which takes place in a natural environment” [2], often mountainous and with considerable differences in height; and where the route winds largely on paths, which are often (but not always) balised.

We generally speak of ultra-trail when the race distance exceeds 42.195 km (26,21 miles), the length of a classic marathon, although the term is used to denote extremely different races. From both a psychological and physiological point of view, competing in a 50 km (31,06 miles) race is markedly different form participating in a 450 km (280 miles) race [3,4]. Ultra-trail races are an extreme sporting challenge: in addition to high training loads, they also require the skills to cope with a wide variety of difficult situations to be completed.

During the competition, the trail runner has to face various challenges, including adverse weather and extreme environmental conditions, or the consequences of sleep deprivation with a relative decline in cognitive abilities and even hallucinations, along with inflammation and muscle pain, metabolic crises, severe fatigue and dehydration [5,6]; These races also involve exposure to unforeseen events such as losing track, encountering wildlife or navigating dangerous or technical passages in the mountains [7].

Due to the extraordinary psychological and physical demands imposed on ultra-trail runners, typical dropout rates can reach 50% and sometimes even exceed this percentage [8].

In psychology the ability to produce adaptation strategies to problems and difficulties, whether extreme or not, is defined as coping skills. Specifically, the coping process is defined as the set of behaviours, thoughts and attitudes that allow an individual to manage the internal and external demands of a stressful situation [9].

The best-known theoretical formulation of coping is the one proposed by Lazarus and Folkman [10] in their “transactional model of stress and coping”, a structure then further developed by Lazarus through the cognitive-motivational-relational theory [11,12]. According to this theoretical framework, an individual perceives as “stressful” those demands from the environment, whether internal or external that exceed their ability to cope effectively with them. In this way, stress turns out to be the result of a transaction between environmental factors and individual abilities [13]. We have already used Lazarus and Folkman’s transactional model of stress in a previous study [14], finding it effective in explaining both psychologically and physiologically mediated phenomena, such as HRV; therefore, we will continue to use this paradigm here too.

In the context of coping strategies applied to ultra-trails, we have found that the distinction between functional and dysfunctional strategies is extremely useful [15]: this is because the coping strategies implemented by the subject are not always effective in solving the problem or reducing emotional distress; on the contrary, they can increase both in the long term, as can happen – for example – when the athlete adopts strategies of anticipation, hypervigilance, or catastrophizing towards a painful symptom or sensation instead of using approaches based on acceptance, rational understanding, or distraction [16].

In the scientific literature, coping strategies have been studied in relation to different sectors of human performance: polar expeditions [17], astronautics [18], marathons [19–21] and ultra-endurance [22–25]. By “ultra-endurance” we refer to sporting events that last longer than six hours and include disciplines such as foot races, ultra-triathlons, ultra-distance swimming, ultra-cycling, and cross-country skiing” [26].

Despite all this, the topic of coping has rarely received attention in the ultra-trail field. Only in recent years, thanks to the growing popularity of the discipline, have a few studies begun to emerge (see for example: [6,27,28]).

What has been missing so far, however, is the development of specific taxonomies addressing both the stressors and the coping strategies used in ultra-trail races of extreme duration. The studies cited focus on ultra-trails of duration significantly shorter than 200 miles, a distance that seems to be a watershed in many respects. Distances exceeding 200 miles involve more days and nights of walking, making the manifestation of various problems more likely, such as changes in weather, the onset of inflammation and medical complications, physical and emotional exhaustion, as well as equipment failure. Furthermore, such long races necessarily require a change in strategy for managing one of the most powerful stressors: sleep deprivation. In these contexts, simple total sleep deprivation strategies are no longer feasible [29]. In other words, sleep becomes a mandatory and urgent necessity, but one that must be managed wisely.

For all these reasons, we focused on a ultra-trail considered to be among the most challenging in the world. The “*Tor des Glaciers” (TDG450)* involves a route of 450 kilometers (280 miles), 32,000 meters of positive difference in altitude and the transit of some passes above 3000 meters above sea level. This course is also without “balises”, it lacks the usual flags, signs, or other markings that help runners navigate and stay on the correct path. The length, the altitude changes, the duration and the severity of the context in which it takes place, with high altitude, technical passages and variable weather, make it suitable for exhaustively examining the coping strategies of ultra-trailers.

The aim of the study was to fill a gap in the literature by developing specific taxonomies that collect, into distinct conceptual categories, both the stressors encountered and coping strategies employed by ultra-trail runners in competitions longer than 200 miles. Our hypothesis was that these taxonomies would be adequate to describe and evaluate the coping strategies used by athletes in a real competition and furthermore, that the coping strategies used by the athletes admitted competing in the *TDG450,* all of them being very experienced, would likely have been largely functional to the context.

## Methods

### Stage A – Elaboration of taxonomies

**Fig 1:**
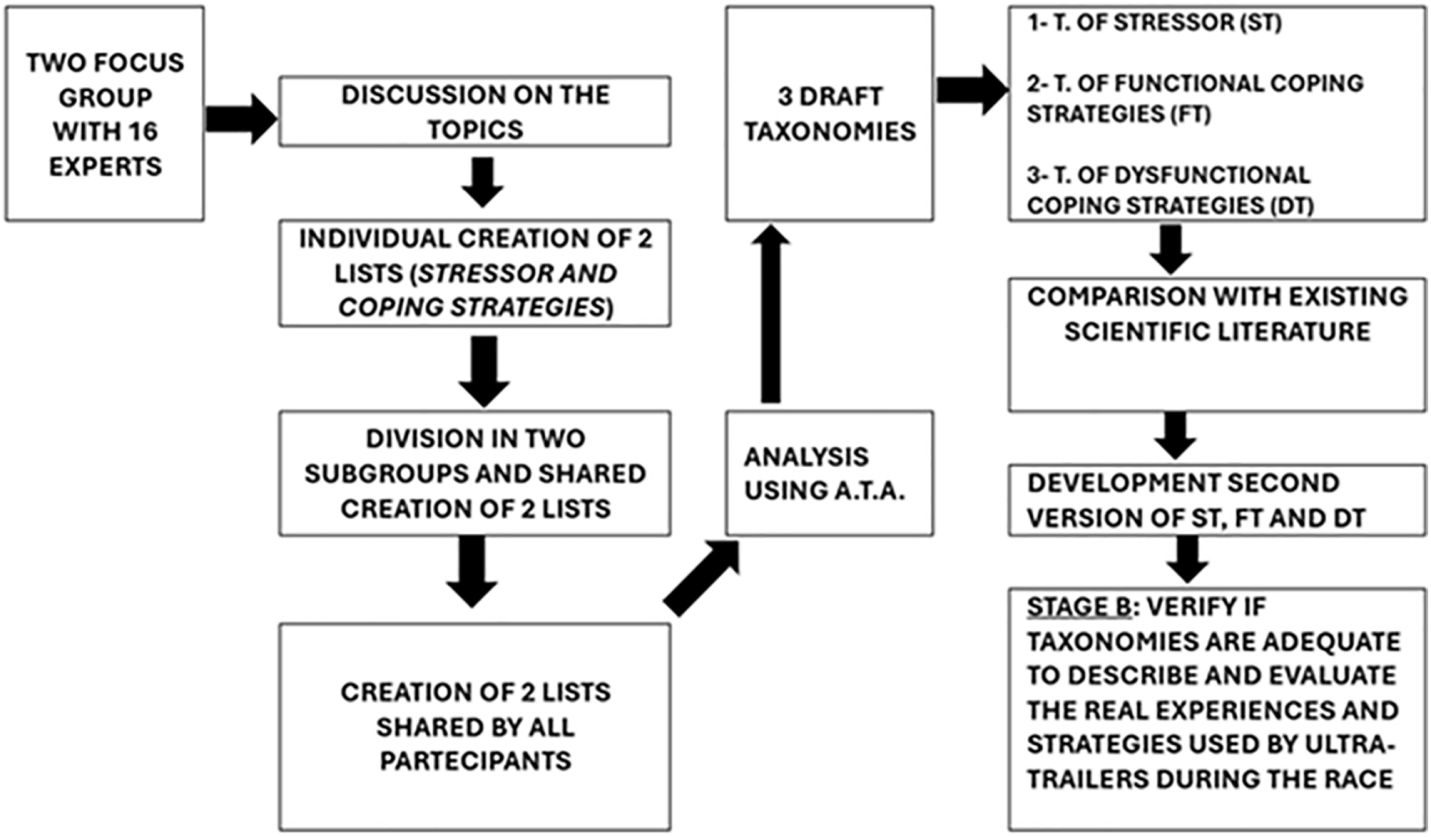
Flowchart of Stage A.

To develop specific taxonomies regarding stressors and coping strategies in races longer than 200 miles, we used two distinct phases: in the first, called “STAGE A” (see Fig 1), to produce a draft of the taxonomies, the first step consisted of identifying a methodology for building and conducting the Focus Groups. We used a methodology called “mini-Delphi” [30]. The mini-Delphi is a simplified variant of the Delphi method, “a technique used to obtain answers to a problem from a group (panel) of independent experts through two or three phases” [31], typically used with a smaller group of experts requiring less time and resources. Two face-to-face Focus Groups were conducted. The inclusion criterion for the Focus Group participants was to be an expert figure who revolved around 200 miles ultra-trail races: athletes, organizers, volunteers, coaches and medical and paramedical staff. The sample was composed according to the logic of judgmental sampling. This phase of the research involved a total of 16 participants (7 females, 9 males age 51,6 ± 6,2) engaged for the various roles within two Italian ultra-trail races: Tor des Geants (330 km, i.e., 205 miles) and TDG450 (450 km, i.e., 280 miles). Potential participants were initially contacted via email with follow-up phone calls made to those who expressed interest. No one refused to participate or dropped out of the study.

The group leader, PT (PsyD), in charge of Psychological Research on Adaptations to Extreme Environments at CERISM, led the Focus Group with methodological support from CG (PhD) and IV (PhD), full professor and researcher at the Research Center in Communication Psychology (PsiCom) at Università Cattolica. The interviewer had previous experience conducting qualitative interviews, having interviewed many ultra-trail runners in previous research projects or while writing books. For this reason, he had prior knowledge of some of the Focus Group participants, particularly the event organizers. Participants were informed in advance that the researchers were conducting a study on certain aspects of ultra-trailers’ mental attitudes and that the results would be published in academic journals. The researchers avoided explicitly stating any hypotheses or expected results so as not to influence the groups’ work. The focus groups were conducted at the headquarters of the company organizing the competition, and aside from the participants and the researcher conducting the interviews, no one else was present. All interviews were audio-recorded with the participants’ consent. Notes were taken during the focus groups to capture contextual information. No repeat interviews were conducted. Each Focus Group lasted a total of two hours.

The rationale of the Focus Group includes three areas of discussion: (a) identification of problems (physical, practical, psychological, i.e., stressors), (b) identification of strategies (functional and dysfunctional, i.e., coping strategies), and (c) identification of factors that influence the development of functional strategies.

Each group had the following agenda divided into four steps:

1. Discussion of the topics present in the rationale of the Focus Group
2. Individual creation of 2 lists on rationale themes (List 1: What do you think are the categories of stressors that a runner could encounter in an ultra-trail? List 2: What are the coping strategies that he could use according to your experience?)
3. Division of the Focus Group participants into two subgroups and shared creation of 2 lists on the rationale themes

Shared creation by all Focus Group participants of two single lists: List 1 and List 2. The shared transcription of the two lists on a flipchart allowed participants to verify and provide feedback on the collected data.

Data saturation was discussed: it was believed that bringing together such a representative group of subjects with diverse viewpoints was in itself a guarantee that the conclusions were based on a sufficiently rich data set. Furthermore, another sign of reaching saturation was the recurrence of the same themes in both focus groups. Regarding the analysis, the authors used Applied Thematic Analysis (ATA), a qualitative method guided by an inductive approach to the analysis of data [32]. It aims to detect the explicit and implicit concepts (i.e., themes) in the data about subjects’ feelings, thoughts, and behaviours to enhance their description and understanding. The detected themes are transformed into codes during the analysis and organised into a codebook. ATA comprises a variety of analytic techniques. Guest, one of the reference authors of this methodology [32], developed the ATA by considering the necessity of providing practical suggestions for conducting the analysis. ATA is suitable for the analysis of textual materials, including Focus Groups. ATA adopts an exploratory analytic approach aiming to explore the phenomenology of stressors and coping strategies relevant to ultra-trailers. Each stressor and each coping strategy identified through the data is treated as a theme that emerges inductively, and each theme is defined based on the data itself. The data were coded by a researcher (IV) under the supervision of a senior researcher (CG) and receiving feedback from the focus group leader (PT) and another qualitative analysis expert (GG). ATLAS.ti software was used to manage the data (Fig.1). It helps to build a coding framework based on the numbered and identified statements provided by the participants.

The initial coding framework, not present in the manuscript, with primary codes consistently linked to quotes from participant interviews, led to the creation of three provisional taxonomies. No secondary or divergent themes emerged in the Focus Groups. A summary of the preliminary results was shared with participants from both groups. Their feedback was noted; and through discussion, two codes were merged into a single one with a more general and comprehensive meaning, compassing the previous code both for functional and dysfunctional strategies. During the discussion, the categories of the three taxonomies were formalized and rationalized to make them as abstract as possible. The provisional taxonomies were then critically revised considering the comparison with other studies in the scientific literature that had examined perceived stressors and coping strategies used in ultra-trail [27–29,33], albeit with reference to shorter races. As discussed in the following section, this critical review allowed us to identify two categories of strategies that had not emerged in the Focus Groups and in the subsequent discussion. This was the second version of the three taxonomies, the one to be tested by interviewing athletes during a real competition.

### Stage B – Application of the taxonomies produced in stage a to real cases to verify their limits and exhaustiveness

In the second phase, “STAGE B”, the taxonomies produced by the Focus Groups were applied to a real case to understand if they were truly exhaustive and effective in explaining the experiences of ultra-trailers. Seven subjects were recruited from the 2024 edition of the TDG450 km race, including six males and one female. They had a mean age of 59.9 ± 3.8 years (range: 54–65 years), a mean stature of 175.6 ± 6.8 cm (range: 163–186 cm), a mean body mass of 68.9 ± 10.5 kg (range: 48–81 kg), and a mean body mass index (BMI) of 22.2 ± 2.1 kg/cm² (range: 18.1–23.9 kg/cm²). All participants were active adults, without cardiovascular diseases or other pathologies limiting physical activity, not professional runners. They had gained some experience in participating in ultra-distance races and all had participated and completed at least one edition of the Tor de Géants 330 (another ultra-trail from the same organizational structure with 330 km and 24.000 meters of altitude difference) in less than 130 hours, this being a requirement to proceed with registration for the TDG450 event. This requirement, requested by the organization, allowed us to abstain from defining additional inclusion criteria for the study, while ensuring certainty regarding the experience and physical conditioning for participation in ultra-distance races of the athletes examined. The average number of years of experience in ultra-trails within the group of participants was 17,28 ± 6,98. Initially, it was decided that all participants would be included in the study, regardless of whether they completed the race.

To collect data during the race, four researchers reached the subjects in the various refuges or life-bases used as refreshment points for the competitors. The maximum interval between data collection, defined in the design phase of the study, was 24 hours; while the minimum was 12 hours. At each control point the questions required by the study were administered in a place free of distractions provided by the organization, near the refuges or life-bases. The entire conversation with the participant was recorded using a mobile phone.

The first question asked was about the greatest difficulty the participant had encountered since the previous interview. During this phase, the athlete was given the opportunity to describe the experience freely, without the researchers interfering with the answer. The next question concerned the strategy used by the athletes to deal with the difficulty encountered, that is, the coping strategy used. Also in this case, the subjects were left completely free to describing the experience without external interference. Subsequently, the recording of this part of data collection was listened to and transcribed verbatim, that is, copying the words spoken exactly, without making any synthesis, by the researchers.

The transcribed material was compared with the previously obtained taxonomies, to verify:

- If the experiences lived in the competition fit within the previously developed taxonomies;
- If new experiences emerged that were not included in the previous taxonomies.

To carry out this analysis, three expert evaluators in the field (all finishers in past editions of the Tor des Geants 330) -different from the four researchers who had conducted the interviews – were employed. Fleiss’s K statistic [34] was used to measure the level of agreement among the three raters in categorizing the interview content according to the three taxonomies. This coefficient ranges from –1–1, where higher values indicate stronger agreement, and negative values reflect systematic disagreement. Along with the kappa value, the associated p-value was calculated to test the null hypothesis that the observed agreement is due to chance. A p-value < 0.05 was considered statistically significant. IBM SPSS Statistics (version 29) was used for the calculations.

We also aimed to measure the ratio of functional coping strategies to dysfunctional ones used by each athlete. This in our opinion could be related to the degree of experience of the subject, even if we cannot demonstrate this in this study. We have defined this measure SCORE F; it is calculated by adding all the functional strategies identified, including those judged by experts to be in fact functional, but which were not included in the first version of the taxonomy (indicated as S.N.A.); and dividing them by the total number of strategies used, therefore also dysfunctional ones.

Both Stage A participants and those interviewed in Stage B provided written consent and assent before participation, and all procedures were approved by CARP (Comitato di approvazione della Ricerca sulla Persona) – University of Verona. Participant recruitment took place from February 7th to the end of March for Phase 1 and from April 10th to July 30th 2024 for Phase 2.

## Results

### Stage A

From the material produced by the Focus Groups and analysed with ATA, the first version of the three taxonomies emerged: the “Perceived Stressors Taxonomy” (ST), including nine distinct macro categories; the “Functional Strategies Taxonomy” (FT), with seven macrocategories became composed of nine macro categories, based on the review of existing literature (see the discussion for more details). Finally, a “Dysfunctional Strategies Taxonomy” (DT), composed of 8 macrocategories.

### Stage B

The interviews carried out during the TDG450 were 40. In Table 1 it is possible to find the overall data on the interviews related to the subjects. Of these interviews, only 34 were used because in some cases the subjects declared that they had not had any difficulty in the stretch travelled in the last 24 hours; or they were in such a prostrate psycho-physical state that the interview did not contain usable material. The expert panel attributed independently the difficulties to the ST. They were able to attribute all the difficulties that have emerged from interview to pre-existing categories; therefore, there was no need to hypothesize the insertion of new categories in ST.

**Table 1:** Demographic data and information obtained from interviews.

SUBJ	GENDER AND AGE	AGE	USABLE INTERVIEW/ TOTAL (n)	F. COPING (n)	N.A.	D. COPING (n)	B.N.C.S	TOTAL JUDGMENTS	SCORE F.	FINISHER/ DID NOT FINISH	REASON FOR WITHDRAWAL	ROUTE COMPLETION PERCENTAGE
1	M	55-59	4/4	7	0	4	1	***12***	0.66	DNF	**Medical problem**	61.4
2	M	65-69	5/9	8	3	1	3	***15***	0.91	FN	**_**	100
3	M	50-54	7/9	18	3	0	0	***21***	1.00	FN	**_**	100
4	M	60-64	1/1	0	0	3	0	***3***	0.00	DNF	**Poor preparation?**	15.1
5	F	60-64	9/9	27	0	0	0	***27***	1.00	FN	**_**	100
6	M	55-59	4/4	12	0	0	0	***12***	1.00	DNF	**Medical problem**	61.4
7	M	60-64	4/4	11	0	1	0	***12***	0.91	DNF	**Failure in passing a time barrier**	51.2
**TOTAL**	***6M, 1F***		***34/40***	***83*** *(81.37%)*	***6*** *(5.88%)*	***9*** *(8.82%)*	***4*** *(3.92%)*	***102*** *(100.00%)*				

***INTERVIEW (n)****, number of interviews for subject;* ***USABLE INTERVIEW****, interviews with usable material;* ***F.COPING****, coping strategies identified in the interview by the panel of experts and traceable back to the categories of the taxonomy;* ***D. COPING****: strategies used by subjects that are considered dysfunctional by experts;* ***TOTAL JUDGMENTS****: total of the judgments expressed on each subject by the experts;* ***B.N.C.S****., Behaviours rated as not corresponding to a coping strategy;* ***FN****, he/she finished the race;* ***DNF****, he didn’t finish the race;* ***N.A.,*** *Strategies deemed Functional but which the raters considered not attributable to any pre-existing category*

**Fig 2:**
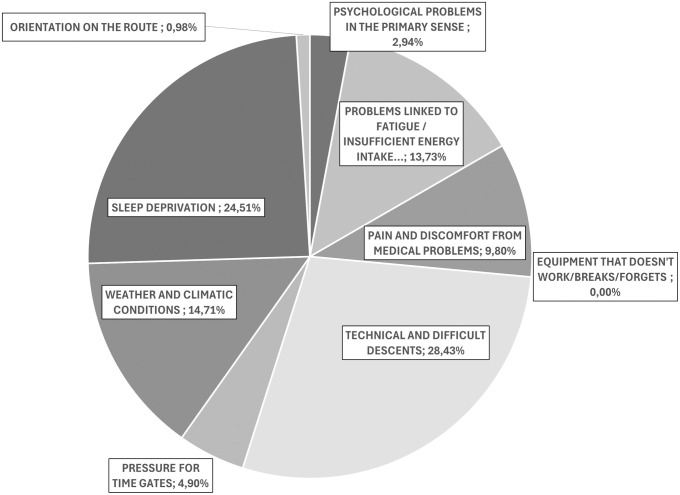
Proportional attribution to stressor’s category produced by the three raters using the first version of ST identified in Stage A.

Fig 2 illustrates the proportional attribution produced by the three raters to category of ST. Fleiss’s K analysis showed a substantial agreement among the raters (K = 0.708; p-value < 0.001). This means that is extremely unlikely that the level of agreement observed is due to chance.

The Coping Strategies identified by the panel of experts were 102, of which 83 (81.37%) were considered attributable to the categories of the FT and 9 (8.82%) to the DT; while 6 (5.88%) Coping Strategies were considered “Functional” but not attributable to any existing category in FT (Table 2).

**Table 2:** Assignment of 7 subjects’ responses to the coping strategies categories.

	Frequency	Percent
1F - DEVELOP CORRECT EXPECTATIONS	2	2,2
2F - FLEXIBILITY AND AWARENESS IN MANAGING PACING, SLEEP AND NUTRITION	30	33,7
3F - PREPARE YOURSELF METICULOUSLY FOR THE SCENARIOS YOU WILL FACE	7	7,9
4F - HAVE PROVEN PROCEDURES TO DEAL WITH THE MOST FREQUENT DIFFICULTIES	10	11,2
5F - EFFECTIVE EMOTIONAL MANAGEMENT OF UNEXPECTED EVENTS	16	18,0
6F - KNOWING HOW TO ASK FOR HELP	9	10,1
7F - ATTENTION AND METACOGNITION	4	4,5
8F - EXPERT MANAGEMENT OF INNER STATES	2	2,2
9F - SET SHORT-TERM GOALS TO DIVIDE THE ENTIRE JOURNEY	3	3,4
*Strategies deemed Functional but which the raters considered not attributable to any pre-existing category*	*6*	*6,7*
	**89**	**100,0**
1D - EXPECTING THE SOLUTION TO COME FROM OUTSIDE	3	33,3
2D - CREATING INCORRECT EXPECTATIONS	1	11,1
4D - GIVE UP ON MANAGING THE PROBLEM	5	55,6
	**9**	**100,0**
*Behaviors that cannot be classified as coping strategies*	*4*	
**TOTAL**	**102**	

**Fig 3:**
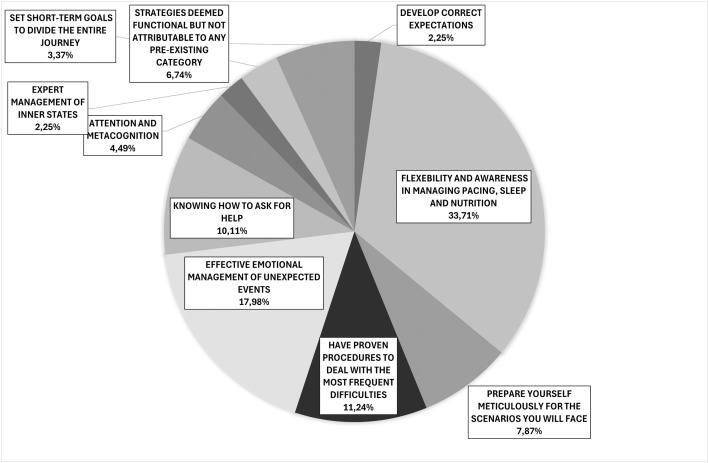
Proportional attribution produced by the three raters to the FT categories as identified in Stage A.

**Fig 4:**
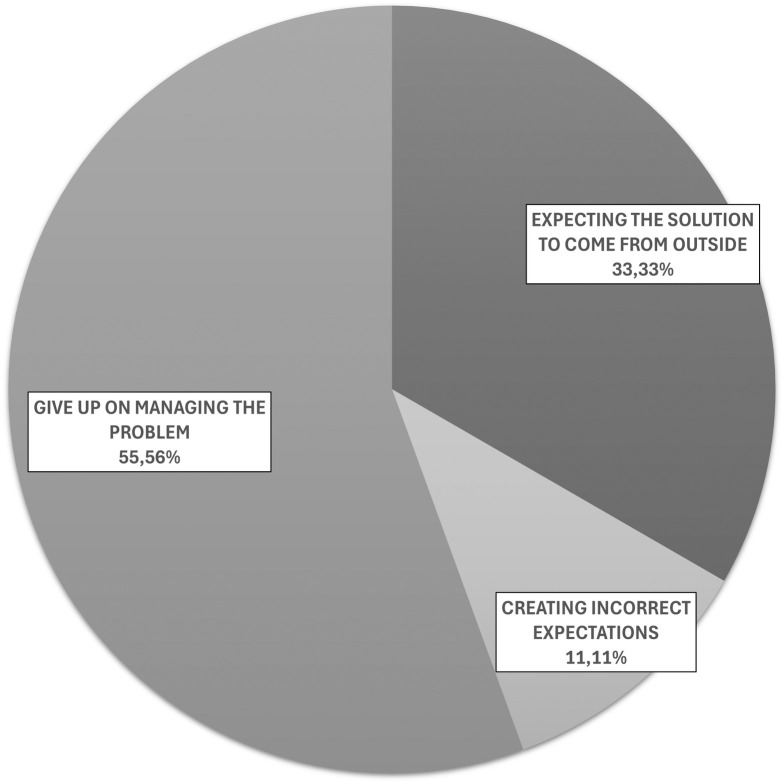
Proportional attribution produced by the three raters to the DT categories as identified in Stage A.

Figures 3 and 4 illustrate the proportional attribution produced by the three raters to the FT and DT categories as identified in Stage A. Fleiss’s K analysis showed a substantial agreement among the raters (K = 0.730; p-value < 0.001). This supports the fact that taxonomies are actually able to “detect” athletes’ coping behaviors.

Four answers by two subjects were considered to be attributable to non-strategic behaviors but simply generated by chance and therefore not classifiable. For example, one subject reported that in a stretch of the route his greatest difficulty had been very strong drowsiness. But his answer to the question “what strategy did you use to face this difficulty?” was: “It happened that I stumbled and fell and hurt myself. The pain kept me awake until here.” One could argue about the fact that his attitude aimed at using pain could be seen as a coping strategy but, in order to have judgments as shareable as possible, some of the experts decided that the event was not intentional; and therefore, not classifiable.

As regards the functional strategies, the athletes (regardless of whether they were finishers or not) obtained a score ranging from 0.66 to 1, with an average of 0.78.

Fig 3 represents the distribution of the raters’ attributions on the FT. It can be noted that all the macro-categories were found within the interviews, although with extremely different percentages of presence: they range from 32.3% of the category “Flexibility and awareness in managing pacing, sleep and nutrition” to 2.2% of “Elaborating correct expectations” and “Expert management of inner states”.

Only three dysfunctional strategies were identified within the interview material. Figure 4 represents the distribution of those strategies.

## Discussion

The results confirmed the hypothesis that the final taxonomies would be able to describe and assess the coping strategies used by athletes in a real competition. Fleiss’s K analysis showed substantial agreement between the raters, supporting the fact that the taxonomies are indeed capable of “detecting” athletes’ coping behaviors. Furthermore, it was demonstrated that—in athletes classified as “experts” based on past competition performances—the use of functional coping strategies prevailed over dysfunctional ones.

### Importance of Experience and Age Effect

Whether this latter result is a coincidence or whether a causal relationship actually exists between “experience” and a more functional use of coping strategies is obviously beyond our ability to demonstrate in this exploratory study and will therefore need to be the subject of further research. If this relationship were demonstrated, it would mean that athletes in these competitions undergo a learning process. In this regard, several studies support the idea that experience, and in particular the number of previous races, is an important predictor of ultra-endurance performance [35–38]. We can hypothesize that, race after race, the athlete, facing ever new problematic situations and difficulties, undergoes a process of learning and optimizing coping strategies. This process may be less evident in ultramarathon competitions that take place in less wild and unpredictable environments than high mountains (for example, road races, however long); and which are characterized by a stage format, which eliminates one of the major stressors (sleep deprivation) as well as the uncertainties of the night [39].

Furthermore, recent findings suggest that the age at which peak performance is achieved in ultramarathons appears to increase with increasing race distance [40,41]. These findings may further confirm that experience is a key factor in success in ultra-endurance; and since the consolidation of learning through experience takes time, the age of peak performance tends to shift forward.. This is also linked to the age of the subjects interviewed in our research, all aged between 50 and 60 years, a phenomenon not uncommon in long races like the TDG450.

### Stressor: “Technical descents” and the “Four big D”

Inside ST, the most represented category (28.43% of responses) was “Technical and difficult Descents”, followed by the “Four Big D”, i.e., the four classic great difficulties of ultra-trail runners: fatigue, pain, sleep deprivation, extreme weather.” In fact, difficulties related to sleep deprivation totalled 24.5%, “Weather and climate conditions” totalled 14.7%; while problems related to fatigue scored 13.72% and the category “Pain and Discomfort from Medical Problems” scored 9.8%.

The result relating to the category “Technical and difficult Descents” is a finding that cannot be generalized to any ultra-trail over 200 miles; rather, it represents a particularity of the Tor des Glacier 450 which includes particularly technical descents, some of which with “via ferrata” sections and which the athlete sometimes has to travel at night or in adverse weather conditions. The difficulty with respect to the descents is not only technical, but above all emotional: some athletes reported feeling anxious or fearful when approaching some of these descents, some of which were “notorious”. This point is interesting because it shows how every external difficulty always secondarily produces an emotional load – although to a different extent depending on the individual: for example, losing orientation or facing a storm becomes terrifying for some; for others it is simply a feeling of annoyance.

In other words, any difficulty that is not psychological in the primary sense (such as the arrival of a storm or loss of orientation) always produces a psychological response, such as anxiety or panic in the scenario mentioned. Sometimes, however, the difficulties are psychological in a primary sense, as Holt also underlines [6]. This refers to stress caused directly by psychological factors, such as facing a competition with wrong expectations, low sense of self-efficacy, or anxiety generated by the possibility of failure. In these cases, thoughts are the direct source of stress, rather than secondary consequences of external difficulties.

It is interesting to note that the category “Psychological problems in a primary sense” is one of the least represented (2.94%). This could be explained by the long experience of the seven ultra-trailers; who, as already specified, had an average previous experience of about 17 years in this type of race. In other words, we could hypothesise that athletes go through – during their career – a process of learning from experience. Through this, they learn to manage their thoughts and emotional responses better or even accept unpleasant thoughts and emotions. Ultimately, this will optimise the choice and use of coping strategies. The complete taxonomy of stressors can be found in the appendix.

### FT: Categories added by reviewing the focus group results in light of the literature on the topic

The seven categories of functional coping that emerged from the Focus Groups and the ATA analysis were compared with the persistent literature on the topic, although it refers to shorter races. The comparison revealed two gaps.

The first concerns the absence in the provisional taxonomy of a very specific category of coping, which we could define: “Setting short-term goals to divide the entire journey”. This is a form of cognitive restructuring in which the athlete chooses to focus on short-term goals, instead of thinking in terms of the entire journey, which can demotivate or cause anxiety [6,42]. It is a form of coping mentioned in the literature, but also often reported in the subjective narratives of athletes. The category was then added to the taxonomy used for the interviews with the seven subjects of the TDG450; and its use was detected in 3.4% of the responses on functional coping strategies.

There is another category of coping strategies that is absent from the initial Focus Group taxonomy but is often reported both anecdotally and in the scientific literature. We could define it as “Expert Management of Internal States”. This means the ability to correctly notice and interpret the meaning of bodily sensations, moods and emotions. Regarding the ability to notice internal information, it has been repeatedly observed in the literature that accessing one’s internal states is a skill that can vary greatly from person to person [43]. Likewise, the ability to interpret internal information – a relevant skill in ultra-trails – also varies from person to person. For example, Clark has noted how anxious people tend to interpret bodily sensations in an alarmist way [44]. During an ultra-trail it is essential for the athlete to be able to evaluate whether the twinges they are feeling are the symptom of cardiac damage or are simply a somatization of anxiety or – again – a general malaise linked to extreme tiredness: while the first case requires rapid action, the other two require the ability to accept the discomfort. “Expert management” therefore means awareness, ability to interpret meaning, acceptance; but also – within certain limits – regulation. According to some authors [33,45,46], for example, mood fluctuations are often related to performance failures in ultrarunning. One of the hypotheses underlying this phenomenon is that severe mood swings force the athlete to make a great effort of self-regulation, taking away more energy; a trap that the expert runner would be able to avoid. We included this category, “Expert interpretation of internal information”, in the final taxonomy that was used for the interviews with the seven TDG450 subjects, and it was detected in 2.2% of the responses regarding functional coping strategies.

### FT: most used strategies in tdg450 interviews

The two categories mentioned above were added to the first version of the taxonomy, produced by the Focus Groups, thanks to the comparison with the existing literature. The seven original categories therefore became nine. And this second version of the taxonomy “Functional Coping Strategies” was used to classify the material that emerged from the interviews with the seven athletes interviewed during the TDG450. Here we will analyse the first three categories that emerged most frequently from the interviews, leaving the others to the complete taxonomy available in the appendix.

The category that appears most frequently in the athletes’ responses is “Flexibility and awareness in managing pacing, sleep and nutrition” (33.7%); it concerns the flexibility in adjusting the pace to the levels of perceived tiredness, to avoid both exhaustion and the failure of time barriers. The same goes for flexibility in eating, finding the right compromise between energy needs and refusal of food due to tiredness. Likewise, sleep management also requires awareness: the athlete cannot sleep at rigidly pre-established rhythms and must have the clarity to understand when a break can no longer be postponed or when instead one can continue a little longer. Having this coping strategy seems to be fundamental for success in the longest ultra-trails.

This skill also involves good contact with the reality of one’s bodily sensations (e.g., “I realized that I was spending too much on that climb and I decided to slow down so as not to exhaust myself”); but, unlike the strategy mentioned above, “Expert management of internal states”, is not only a matter of interpretation, but also of active monitoring of sensations in relation to the effort to be made. Brick [47] also makes a similar distinction: speaking about attentional strategies during endurance activity, he distinguishes between “Internal sensory Monitoring”, focused on internal sensations and comparable to the “Expert Management of inner states”; and “Active Self-Regulation Strategies”, in which attention is paid to rhythm, cadence and pace in relation to effort.

The second and third most frequently detected strategies in the interviews were “Effective emotional management of unexpected events” (18.0%) and “Have proven procedures to deal with the most frequent difficulties” (11.2%) respectively.

“Effective emotional management of unexpected events” refers to the ability to manage adverse emotional states (anxiety, demotivation, panic) – and possibly use one’s experience to find solutions – when situations occur that are unpredictable because they are statistically rare or unpredictable: for example, an athlete referred to an episode where he had to rescue other competitors who were panicking on a stretch of “via ferrata”. Other examples may be those relating to losing orientation in sections that were presumed to be well known or finding technical descents in particularly dangerous conditions due to unusual climatic conditions.

The category “Have proven procedures to deal with the most frequent difficulties” refers instead to the possession of procedures or protocols capable of dealing with frequent and foreseeable difficulties. For example: having a protocol and material to treat a blister; knowing how to behave when you get lost; knowing what to do in case of cramps, etc. The three categories described above alone collect 63% of the functional strategies identified in the interviews.

### FT: Coping strategies not attributable to pre-existing categories

It is important to note that the three raters were unable to attribute to FT the 6.7% of the answers to the question “what strategy did you use to deal with this difficulty?”. For example, one ultra-trailer reported that the greatest difficulty he had faced in the previous 24 hours was a state of paralyzing drowsiness; it lasted from the last refuge he encountered and for the subsequent climb to a pass; and for the very long descent that followed. When asked what strategy he had implemented to deal with this difficulty, he replied: “I persevered”. In this case, all three expert raters were unable to place the strategy within the categories of FT, although they agreed that it was a functional strategy. The other answers that the raters were unable to classify also referred to concepts such as “I did not give up and continued anyway”, “I held on”, etc.

All this led us to observe that the initial taxonomy that emerged from the Focus Groups lacks a category of coping strategies focused on the use of self-regulation and volitional skills; we refer to the ability to “fully engage and persevere in the absence of immediate gratification” which is connected to the concept of resilience and some aspects of motivation [48]. Research has shown that self-regulation is a key resource in endurance performance, being involved both in maintaining pace and in decision-making processes regarding the continuation or interruption of the race (see for example the critical review by Hyland-Monks [49]). It is conceivable that a high self-regulation capacity represents one of the functional coping strategies usually used by athletes in extreme endurance races. Research has shown that self-regulation applied to endurance disciplines is a limited resource, the exhaustion of which determines a decline in performance [50,51]; but also how it can be increased with training and experience [52]. For these reasons we decided to aggregate these 6.7% of unattributed responses by addressing them in the category “Willpower and self-regulation skills”. The complete taxonomy of coping strategies is available in the appendix.

### Dysfunctional coping strategies

Even after the comparison phase with existing literature, the DT categories that emerged from the Focus Groups remained eight. Examining the interview material, the expert panel defined only 8.82% of the total coping strategies as “dysfunctional”. As previously underlined, this “rarefied” presence of dysfunctional strategies could be linked to the fact that the competitors were all experts. A future development of the research could be to analyse the differences in the use of coping strategies between “beginners” and “experts”. This is to verify, as it would seem intuitive, that beginners use a higher percentage of dysfunctional strategies than experts.

Within this 8.82%, the attribution involved only three categories from the eight that emerged from the Focus Group phase. “Giving up on problem management”, with 55.6%, is the most represented category. A typical example of this behavior is the athlete who states “to have two strategies to combat drowsiness: either wash the face with cold water or stop for a micro-sleep and to have given up both, remaining passive in the face of drowsiness until the moment of withdrawal from the race”.

The dysfunctional strategy “Expecting the solution to come from outside” totalled 33.3% of attributions. A typical example of this behavior could be the competitor who does not check his route on the GPS because he counts on those who precede him in sight to do it for him (without thinking that they could also be wrong). Finally, “Creating incorrect expectations” (11.1%) is in third place. An example could be the runner who has clearly underestimated the degree of fatigue or psychological discomfort he will encounter during the race; and this leads him to abandon the competition. The complete taxonomy accompanied by examples can be found in the appendix.

### Analysis of functional coping score

The score in the use of functional strategies is high for all subjects (finisher or not) who have exceeded 50% of the total course, ranging from 0.66 to 1. It is relevant to note that the DNF subject who withdrew after having covered only 15% of the course recorded a score of 0. The hypothesis that there is a causal correlation between lower scores and withdrawal is fascinating: between lower use of functional strategies and the fact of not finishing the race. However, the three cases of withdrawal that occurred after the halfway point are due to causes that could be fortuitous and not necessarily linked only to the use of dysfunctional coping strategies: medical problems in two cases and failure to pass a time barrier due to a delay of 60 minutes after 88 hours of racing. Therefore, any correlation between a high level of functional strategy use and success in ultra-trail running needs to be demonstrated differently.

## Limits

A limitation of the research is that the initial categories of the taxonomies, generated by the Focus Groups, are experiential. Having then made a comparison with the scientific literature had the aim of making them more abstract and rationalizing them, but the objective was certainly achieved in an imperfect way. In fact, there are conceptual overlaps between the functional coping categories; just think in FT of the theme of attention, which recurs in the categories “Attention and Metacognition”, “Expert management of Inner states” and “Flexibility and Awareness in Managing pacing..”. Another limitation of the taxonomies, already highlighted in the discussion, is the fact that they were obtained by processing the experiences of two similar and completely peculiar races; and the results (at least in terms of relative weight of the specific categories) are not always generalizable to all ultratrails. Proof of this is the fact that the greatest perceived stress factor was found to be “technical and challenging descents”, which obviously does not apply to most ultratrails. A third limitation is the size of the sample used (7 athletes, with a gender difference): a statistic based on such a small sample is not able to detect coherent trends. For this reason and for the reasons already listed on the possible accidentality of withdrawals, we avoided using conclusions based on the Score statistic.

## Conclusions and future developments

The aim of the study was to fill a gap in the literature to arrive at the development of specific taxonomies that collect in distinct conceptual categories the stressors that athletes must face in ultratrail races longer than 200 miles. The result is three specific taxonomies that – as we have verified – allow the evaluation of coping strategies used by athletes in a real competition. They can be used as a starting point for further research or to build questionnaires, which would allow to involve a greater number of athletes Furthermore, the taxonomies can be used as a training tool to educate inexperienced runners about the challenges they may encounter in ultra-trails, and also to raise awareness and make them aware of the coping strategies they employ during competitions. The athletes examined appeared to possess coping strategies that were functional to the context and, probably, had developed them over the years of practice, being all very experienced. Future research could include analysing functional coping strategy scores between athletes with a long history of experience and athletes with significantly less experience; if a correlation between high scores and years of experience is verified, this would definitively demonstrate that the use of functional coping strategies is learned through experience.

## Supporting information

S1 Table Taxonomy of perceived stressors (ST). (PDF)

S2 Table Taxonomy of dysfunctional coping strategies (DT). (PDF)

S3 Table Taxonomy of functional coping strategies (FT). (PDF)

S4 Table Fleiss Kappa – perceived stressors taxonomy. (PDF)

S5 Table Fleiss Kappa – functional and dysfunctional strategies taxonomy. (PDF)
