# Correction: It’s not difficulty that matters, but strategy: Perceived stressor, functional and dysfunctional coping strategies in ultra-trails of extreme duration

**DOI:** 10.1371/journal.pone.0342930

**Published:** 2026-02-12

**Authors:** The PLOS One Staff

There are errors in the author affiliations. The correct affiliations are as follows:

Pietro Trabucchi^1^, Barbara Pellegrini^1,2^, Aldo Savoldelli^1,3^, Gianandrea Giacoma^4^, Ilaria Vergine^5^, Carlo Galimberti^5^, Sara Garofalo^6^, Federico Schena^1,7^

1 CERISM, Centro Ricerca Interuniversitario Sport Montagna e Salute, Rovereto, Italy, 2 Department of Engineering for Innovation in Medicine, University of Verona, Verona, Italy, 3 CIBIO, Department of Cellular, Computational and Integrative Biology; University of Trento, Trento, Italy, 4 Independent researcher, Milano, Italy, 5 Research Center in Communication Psychology (PsiCom), Università Cattolica, Milan, Italy, 6 Department of Psychology, University of Bologna, Bologna, Italy, 7 Department of Neuroscience, Biomedicine and Movement, University of Verona, Verona, Italy.

The publisher apologizes for the error.
