# Supplementary material for: It’s not difficulty that matters, but strategy: Perceived stressor, functional and dysfunctional coping strategies in ultra-trails of extreme duration
Source: PLoS One. 2025 Sep 12;20(9):e0332058. doi: 10.1371/journal.pone.0332058 (PMC12431310; doi:10.1371/journal.pone.0332058)
Supplement: S1 Table — (PDF) [file pone.0332058.s001.pdf]

Supporting Information Table 1. Taxonomy of Perceived Stressors (ST)

GENERAL CATEGORIES OF TYPICAL PERCEIVED STRESSORS IN ULTRA-TRAILS (not in hierarchical order).	***DESCRIPTION AND PRACTICAL EXAMPLES***
1. ORIENTATION ON THE ROUTE	*Difficulties related to orienting oneself on the race track. For example: absence of beacons on a track that instead provides them; absence of GPS signal; difficulty in orienting oneself due to the weather.*
2. SLEEP DEPRIVATION	*Difficulties related to sleep deprivation, motivated by the choice not to sleep in order to pass the time gates and finish the race within the established time. For example: presence of hallucinations, decline in cognitive performance, physical inability to continue, falling asleep during the race, extreme tiredness.*
3- WEATHER AND CLIMATIC CONDITIONS	*Difficulties related to adverse weather conditions that add problems to progression, orientation and/or related to thermoregulation. Example: fog, snow, presence of icy stretches, rain, blizzard, cold, heat, mugginess, solar radiation at high altitude, wind.*
4- PRESSURE FOR TIME GATES	*Difficulties related to the psychological stress caused by the prospect of not passing the pre-established time barriers in time, present at some points of the route (they prevent participants who do not pass them by the closing time from finishing the race because they are too slow). This pressure can cause subjective discomfort such as anxiety, but also lead to opting for dysfunctional tactical choices (neglecting breaks and refreshments, incorrect pace, etc.).*
5- TECHNICAL AND DIFFICULT DESCENTS	*Difficulties related to the exposure, steepness, risk of falling or slipping of some descents, as well as their cost in terms of time spent and mental energy/cognitive resources used to overcome them.*
6- EQUIPMENT THAT DOESN'T WORK/BREAKS/FORGETS	*Difficulties related to clothing or equipment. For example, a broken pole, wrong socks, dead GPS, equipment left at a refreshment point.*
7- PAIN AND DISCOMFORT FROM MEDICAL PROBLEMS (	*Difficulties relating to the perception of discomfort and/or pain resulting from trauma or pathologies (such as inflammation, muscle damage, heart damage, cramps, blisters, dehydration, etc.) that require medical or nursing or physiotherapy care.*
8- PROBLEMS LINKED TO FATIGUE / INSUFFICIENT ENERGY INTAKE/ /METABOLIC CRISES/DEHYDRATION	*Difficulties related to the perception of general fatigue, exhaustion or muscle weakness that can be generated by an energy and/or fluid deficit; or by lack of training, limits in the durability of the subject. For example: metabolic crises, lack of energy, exhaustion, extreme fatigue.*
9- PSYCHOLOGICAL PROBLEMS IN THE PRIMARY SENSE	*Difficulties and stress caused directly by psychological factors – while the previous factors cause stress or psychological discomfort as a secondary consequence (e.g. anxiety because I am lost, or mental confusion from sleep deprivation). Examples of psychological difficulties in the primary sense: incorrect expectations, low sense of self-efficacy, anxiety related to performance, loss of control, intolerance to frustration, fear of disappointing someone, comparison with other competitors.*
