# Supplementary material for: It’s not difficulty that matters, but strategy: Perceived stressor, functional and dysfunctional coping strategies in ultra-trails of extreme duration
Source: PLoS One. 2025 Sep 12;20(9):e0332058. doi: 10.1371/journal.pone.0332058 (PMC12431310; doi:10.1371/journal.pone.0332058)
Supplement: S2 Table — (PDF) [file pone.0332058.s002.pdf]

Supporting Information Table 2. Taxonomy of Dysfunctional Coping Strategies (DT)

GENERAL CATEGORIES OF DYSFUNCTIONAL COPING STRATEGIES IN ULTRA-TRAILS (not in hierarchical order).	DESCRIPTION AND PRACTICAL EXAMPLES
1- EXPECTING THE SOLUTION TO COME FROM OUTSIDE	*The ultra trailer does not act proactively but waits for her/his problems to be solved by others (family members, organizers and volunteers, other competitors). For example: the competitor does not manage the rest times or does not "restock his backpack" at the life bases because he waits for his family members to do it; the trailer does not control the route because he tacitly counts on those preceding him not to make a mistake.*
2- CREATING INCORRECT EXPECTATIONS	*The ultra trailer creates unrealistic and unsustainable expectations about the travel times, the level of fatigue he will experience, his ability to manage difficult situations. Examples: the ultra trailer expects the race to be similar to a marathon and is not aware that it is necessary to have knowledge about the mountains; ignoring the degree of fatigue or psychological discomfort that one will encounter.*
3- DENY OR IGNORE THE PROBLEM	*The ultra trailer denies the presence of a problem. Ex: the trailer ignores the bad weather that is approaching or the inflammation in the knee.*
4- GIVE UP ON MANAGING THE PROBLEM	*The ultra trailer gives up on dealing with the problem by experiencing events with a sense of impotence to the extreme of finding oneself in a state of "Freezing". Ex: ".. under the Fenetre di Tzan I found a girl who was crying because she couldn't find any more flags. She was in total panic. She had completely let herself go, she was just crying.."*
5- VENT NEGATIVE EMOTIONS ON OTHERS	*The ultra trailer does not seek solutions to problems but simply unloads the resulting discomfort on others in the form of aggression or whining. For example, blaming the organizers if someone loses their way, or venting their discomfort in an aggressive way on the volunteers.*
6 SEEKING EMOTIONAL COMFORT AS AN END IN ITSELF	*The ultra trailer unconsciously asks others to provide emotional comfort and psychological closeness, not taking action to resolve the problem or using a problem as a pretext to initiate interaction.*<br>*Ex: some trailers call the organizers during the race exaggerating a problem such as being lost that they could solve by themselves - only because they actually want to feel someone close*
7-WITHDRAWING FROM THE RACE	*The ultra trailer chooses retirement - although it is not inevitable - as a solution to the difficulties it is encountering, rather than committing to resolving them.*
8- PLAYING THE VICTIM AND CREATING ALIBIS	*The ultra trailer constructs a narrative that presents him as a victim and attributes the failure entirely to external factors, instead of solving the problem.*
