# Supplementary material for: It’s not difficulty that matters, but strategy: Perceived stressor, functional and dysfunctional coping strategies in ultra-trails of extreme duration
Source: PLoS One. 2025 Sep 12;20(9):e0332058. doi: 10.1371/journal.pone.0332058 (PMC12431310; doi:10.1371/journal.pone.0332058)
Supplement: S3 Table — (PDF) [file pone.0332058.s003.pdf]

Supporting Information Table 3. Taxonomy of Functional Coping Strategies (FT)

GENERAL CATEGORIES OF FUNCTIONAL COPING STRATEGIES IN ULTRA-TRAILS (not in hierarchical order).	***DESCRIPTION AND PRACTICAL EXAMPLES***
1- DEVELOP CORRECT EXPECTATIONS	*The ultra trailer has realistic and sustainable expectations. He knows what to expect from the mountain environment and realistically estimates the travel times or levels of fatigue he will experience. These expectations can be the fruitful result of a long experience of the mountain environment and trail races. Example:" I know that that climb will be very tiring both for the difference in altitude and for the time of day, when the sun beats down on the valley. So I will keep a slower pace than usual".*
2- FLEXIBILITY AND AWARENESS IN MANAGING PACING, SLEEP AND NUTRITION	*The ultratrailer has flexibility in managing the pace with which she/he runs the race, adapting it to her/his sensations of fatigue and the needs of the time barrier. This same flexibility and awareness is used towards sleep (she/he tries to be aware of when she/he can still postpone the stops and instead when he must stop and for how long). He/she feeds and hydrates regularly.*
3- PREPARE YOURSELF METICULOUSLY FOR THE SCENARIOS YOU WILL FACE	*The ultra trailer prepares himself meticulously, to the best of his ability and with care for the race in all its aspects (physical, psychological and related to the equipment). Example: train regularly following shared methodologies and criteria, learn about the equipment and try it in advance, try the route where possible.*
4- HAVE PROVEN PROCEDURES TO DEAL WITH THE MOST FREQUENT DIFFICULTIES	*The ultratrailer has procedures or protocols to deal with frequent and predictable difficulties. For example: having a protocol and material to treat a blister; knowing how to manage clothing in extreme cold; knowing what to do in case of cramps, etc.*
1- EFFECTIVE EMOTIONAL MANAGEMENT OF UNEXPECTED EVENTS	*When unpredictable situations arise, the ultra-trailer is able to manage adverse emotional states (anxiety, demotivation, panic) and knows how to use his experience to find solutions. For example: encountering unusual weather conditions (verglas on the path) on an a dangerous section of trail, he keeps calm and discovers that he can bypass that stretch by going to another side.*
6- KNOWING HOW TO ASK FOR HELP	*The ultra trailer knows how to ask for help effectively, identifying competent people who can help him and formulating his request appropriately. He does not confuse the search for emotional comfort as an end in itself with a specific request for help on a concrete problem.*
7- ATTENTION AND METACOGNITION	*During the race, the ultratrailer knows how to adjust his attention according to the needs of the moment; he knows how to concentrate totally when the route requires it, but he is also able to distract himself using his own thoughts or environmental stimuli when the long hours of walking become boring. Furthermore, by directing attention to one's own thought processes and experiences, one is able to increase awareness of how one manages difficulties. This allows him to learn from experience and be increasingly effective in managing himself during the race. E.g.: I had to concentrate totally on the descent because it was dangerous.*

8-	EXPERT MANAGEMENT OF INNER STATES	*The ultra-trailer can expertly interpret bodily sensations, thus avoiding the anxious interpretation of simple body signals and knowing how to distinguish, without underestimating them, when they are symptoms of real pathologies.*
9-	SET SHORT-TERM GOALS TO DIVIDE THE ENTIRE JOURNEY	*The ultratrailer is able to cognitively reframe the huge route (and the emotional sensation that derives from it) in small pieces, focusing each time only on the one closest to him.*
10-	WILLPOWER AND SELF-REGULATION SKILLS	*In unpleasant situations and without an immediate solution, the ultratrailer continues to engage without losing motivation.*
