# Supplementary material for: It’s not difficulty that matters, but strategy: Perceived stressor, functional and dysfunctional coping strategies in ultra-trails of extreme duration
Source: PLoS One. 2025 Sep 12;20(9):e0332058. doi: 10.1371/journal.pone.0332058 (PMC12431310; doi:10.1371/journal.pone.0332058)
Supplement: S4 Table — (PDF) [file pone.0332058.s004.pdf]

Fleiss Kappa – Perceived Stressors Taxonomy

Overall Kappa

	Kappa	Asymptotic Standard Error	Z	P Value	Lower 95% Asymptotic CI Bound	Upper 95% Asymptotic CI Bound
Overall	,708	,046	15,233	,000	,617	,799

Kappas for Individual Categories

Rating Category	Conditional Probability	Kappa	Asymptotic Standard Error	Z	P Value	Lower 95% Asymptotic CI Bound	Upper 95% Asymptotic CI Bound
1	1,000	1,000	,099	10,100	,000	,806	1,194
2	,857	,834	,099	8,427	,000	,640	1,028
3	,500	,446	,099	4,501	,000	,252	,640
5	,828	,759	,099	7,666	,000	,565	,953
6	,800	,790	,099	7,975	,000	,596	,984
7	,600	,531	,099	5,363	,000	,337	,725
8	,840	,788	,099	7,959	,000	,594	,982
9	,000	-,010	,099	-,100	,920	-,204	,184

Categories:

1 - PSYCOLOGICAL PROBLEMS IN THE PRIMARY SENSE

2 - PROBLEMS LINKED TO FATIGUE / INSUFFICIENT ENERGY INTAKE…

3 - MEDICAL PROBLEMS

5 - TECHNICAL AND DIFFICULT DESCENTS

6 - PRESSURE FOR THE GATES

7 - WEATHER AND CLIMATIC CONDITIONS

8 - SLEEP DEPRIVATION

9 - ORIENTATION ON THE ROUTE

Weighted Kappa – Perceived Stressors Taxonomy

Cohen's Weighted Kappa

Ratings	Weighted Kappa[a]	Asymptotic Std. Error[b]	Asymptotic z[c]	Asymptotic Sig.	95% Asymptotic Confidence Interval Lower Bound	95% Asymptotic Confidence Interval Upper Bound
Expert A - Expert B	,693	,099	5,957	<,001	,499	,887
Expert A - Expert C	,806	,082	6,914	<,001	,645	,967
Expert B - Expert C	,717	,109	6,136	<,001	,503	,931

a. The estimation of the weighted kappa uses linear weights.

b. Value does not depend on either null or alternative hypotheses.

c. Estimates the asymptotic standard error assuming the null hypothesis that weighted kappa is zero.

Expert A - Expert B

		Expert B								
		1	2	3	5	6	7	8	9	Total
Expert A	1	1	0	0	0	0	0	0	0	1
	2	0	4	0	0	0	0	0	0	4
	3	0	0	1	1	1	1	1	0	5
	5	0	0	0	8	0	1	0	0	9
	6	0	0	0	0	1	0	0	0	1
	7	0	1	0	2	0	2	0	0	5
	8	0	0	0	0	0	1	7	0	8
	9	0	0	0	0	0	0	1	0	1
Total		1	5	1	11	2	5	9	0	34

Categories:

1 - PSYCOLOGICAL PROBLEMS IN THE PRIMARY SENSE

2 - PROBLEMS LINKED TO FATIGUE / INSUFFICIENT ENERGY INTAKE…

3 - MEDICAL PROBLEMS

5 - TECHNICAL AND DIFFICULT DESCENTS

6 - PRESSURE FOR THE GATES

7 - WEATHER AND CLIMATIC CONDITIONS

8 - SLEEP DEPRIVATION

9 - ORIENTATION ON THE ROUTE

Expert A - Expert C

		Expert C								
		1	2	3	5	6	7	8	9	Total
Expert A	1	1	0	0	0	0	0	0	0	1
	2	0	4	0	0	0	0	0	0	4
	3	0	0	3	1	1	0	0	0	5
	5	0	0	1	7	0	1	0	0	9
	6	0	0	0	0	1	0	0	0	1
	7	0	0	0	1	0	4	0	0	5
	8	0	1	0	0	0	0	7	0	8
	9	0	0	0	0	0	0	1	0	1
Total		1	5	4	9	2	5	8	0	34

Categories:

1 - PSYCOLOGICAL PROBLEMS IN THE PRIMARY SENSE

2 - PROBLEMS LINKED TO FATIGUE / INSUFFICIENT ENERGY INTAKE…

3 - MEDICAL PROBLEMS

5 - TECHNICAL AND DIFFICULT DESCENTS

6 - PRESSURE FOR THE GATES

7 - WEATHER AND CLIMATIC CONDITIONS

8 - SLEEP DEPRIVATION

9 - ORIENTATION ON THE ROUTE

Expert B - Expert C

		Expert C							
		1	2	3	5	6	7	8	Total
Expert B	1	1	0	0	0	0	0	0	1
	2	0	4	0	0	0	1	0	5
	3	0	0	1	0	0	0	0	1
	5	0	0	1	9	0	1	0	11
	6	0	0	0	0	2	0	0	2
	7	0	0	1	0	0	3	1	5
	8	0	1	1	0	0	0	7	9
Total		1	5	4	9	2	5	8	34

Categories:

1 - PSYCOLOGICAL PROBLEMS IN THE PRIMARY SENSE

2 - PROBLEMS LINKED TO FATIGUE / INSUFFICIENT ENERGY INTAKE…

3 - MEDICAL PROBLEMS

5 - TECHNICAL AND DIFFICULT DESCENTS

6 - PRESSURE FOR THE GATES

7 - WEATHER AND CLIMATIC CONDITIONS

8 - SLEEP DEPRIVATION

9 - ORIENTATION ON THE ROUTE
