# Supplementary material for: It’s not difficulty that matters, but strategy: Perceived stressor, functional and dysfunctional coping strategies in ultra-trails of extreme duration
Source: PLoS One. 2025 Sep 12;20(9):e0332058. doi: 10.1371/journal.pone.0332058 (PMC12431310; doi:10.1371/journal.pone.0332058)
Supplement: S5 Table — (PDF) [file pone.0332058.s005.pdf]

Fleiss Kappa – Functional and Dysfunctional Strategies Taxonomy

Overall Kappa

	Kappa	Asymptotic Standard Error	Z	P Value	Lower 95% Asymptotic CI Bound	Upper 95% Asymptotic CI Bound
Overall	,730	,044	16,605	,000	,644	,816

Kappas for Individual Categories

Rating Category	Conditional Probability	Kappa	Asymptotic Standard Error	Z	P Value	Lower 95% Asymptotic CI Bound	Upper 95% Asymptotic CI Bound
1D	,333	,310	,105	2,944	,003	,104	,517
1F	,500	,489	,105	4,636	,000	,282	,695
2D	,000	-,011	,105	-,107	,915	-,218	,195
2F	,867	,800	,105	7,589	,000	,593	1,007
3F	,714	,690	,105	6,548	,000	,484	,897
4D	,333	,310	,105	2,944	,003	,104	,517
4F	,900	,888	,105	8,420	,000	,681	1,094
5F	,813	,772	,105	7,323	,000	,565	,979
6F	,778	,753	,105	7,144	,000	,546	,960
7F	,750	,738	,105	7,005	,000	,532	,945
8F	,500	,489	,105	4,636	,000	,282	,695
9F	1,000	1,000	,105	9,487	,000	,793	1,207

Categories:

1D - EXPECTING THE SOLUTION TO COME FROM OUTSIDE

1F - DEVELOP CORRECT EXPECTATIONS

2D - CREATING INCORRECT EXPECTATIONS

2F - FLEXIBILITY AND AWARENESS IN MANAGING PACING, SLEEP AND NUTRITION

3F - PREPARE YOURSELF METICULOUSLY FOR THE SCENARIOS YOU WILL FACE

4D - GIVE UP ON MANAGING THE PROBLEM

4F - HAVE PROVEN PROCEDURES TO DEAL WITH THE MOST FREQUENT DIFFICULTIES

5F - EFFECTIVE EMOTIONAL MANAGEMENT OF UNEXPECTED EVENTS

6F - KNOWING HOW TO ASK FOR HELP

7F - ATTENTION AND METACOGNITION

8F - EXPERT MANAGEMENT OF INNER STATES

9F - SET SHORT-TERM GOALS TO DIVIDE THE ENTIRE JOURNEY

Weighted Kappa – Functional and Dysfunctional Strategies Taxonomy

Cohen's Weighted Kappa

Ratings	Weighted Kappa[a]	Asymptotic Std. Error[b]	Asymptotic z[c]	Asymptotic Sig.	95% Asymptotic Confidence Interval Lower Bound	95% Asymptotic Confidence Interval Upper Bound
Expert A - Expert B	,532	,138	4,599	<,001	,261	,803
Expert A - Expert C[d]	,787	,105	6,893	<,001	,581	,993
Expert B - Expert C	,660	,129	5,631	<,001	,408	,913

[a.] The estimation of the weighted kappa uses linear weights.

[b.] Value does not depend on either null or alternative hypotheses.

[c.] Estimates the asymptotic standard error assuming the null hypothesis that weighted kappa is zero.

[d.] Categories are determined by observed values and values for which value labels are defined.

Expert A - Expert B

		Expert B												
		1D	1F	2D	2F	3F	4D	4F	5F	6F	7F	8F	9F	Total
Expert A	1D	0	0	0	0	0	0	0	0	0	0	0	0	0
	1F	0	0	0	0	0	0	0	0	0	1	0	0	1
	2D	0	0	0	0	0	0	0	0	0	0	0	0	0
	2F	0	0	0	8	0	1	0	0	0	0	0	0	9
	3F	0	0	0	1	1	0	0	0	0	0	0	0	2
	4D	0	0	1	0	0	0	0	0	0	0	0	0	1
	4F	0	0	0	0	0	0	3	0	0	0	0	0	3
	5F	0	0	0	1	1	0	0	5	0	0	0	0	7
	6F	2	0	0	0	0	0	0	0	2	0	0	0	4
	7F	0	0	0	0	0	0	0	0	0	1	0	0	1
	8F	0	0	0	0	0	0	1	0	0	0	0	0	1
	9F	0	0	0	0	0	0	0	0	0	0	0	1	1
Total		2	0	1	10	2	1	4	5	2	2	0	1	30

Categories:

1D - EXPECTING THE SOLUTION TO COME FROM OUTSIDE

1F - DEVELOP CORRECT EXPECTATIONS

2D - CREATING INCORRECT EXPECTATIONS

2F - FLEXIBILITY AND AWARENESS IN MANAGING PACING, SLEEP AND NUTRITION

3F - PREPARE YOURSELF METICULOUSLY FOR THE SCENARIOS YOU WILL FACE

4D - GIVE UP ON MANAGING THE PROBLEM

4F - HAVE PROVEN PROCEDURES TO DEAL WITH THE MOST FREQUENT DIFFICULTIES

5F - EFFECTIVE EMOTIONAL MANAGEMENT OF UNEXPECTED EVENTS

6F - KNOWING HOW TO ASK FOR HELP

7F - ATTENTION AND METACOGNITION

8F - EXPERT MANAGEMENT OF INNER STATES

9F - SET SHORT-TERM GOALS TO DIVIDE THE ENTIRE JOURNEY

Expert A - Expert C

		Expert C												
		1D	1F	2D	2F	3F	4D	4F	5F	6F	7F	8F	9F	Total
Expert A	1D	0	0	0	0	0	0	0	0	0	0	0	0	0
	1F	0	1	0	0	0	0	0	0	0	0	0	0	1
	2D	0	0	0	0	0	0	0	0	0	0	0	0	0
	2F	0	0	0	9	0	0	0	0	0	0	0	0	9
	3F	0	0	0	0	2	0	0	0	0	0	0	0	2
	4D	0	0	0	0	0	2	0	0	0	0	0	0	2
	4F	0	0	0	0	0	0	3	0	0	0	0	0	3
	5F	0	0	0	2	1	0	0	4	0	0	0	0	7
	6F	1	0	0	0	0	0	0	0	3	0	0	0	4
	7F	0	0	0	0	0	0	0	0	0	1	0	0	1
	8F	0	0	0	0	0	0	0	0	0	0	1	0	1
	9F	0	0	0	0	0	0	0	0	0	0	0	1	1
Total		1	1	0	11	3	2	3	4	3	1	1	1	31

Categories:

1D - EXPECTING THE SOLUTION TO COME FROM OUTSIDE

1F - DEVELOP CORRECT EXPECTATIONS

2D - CREATING INCORRECT EXPECTATIONS

2F - FLEXIBILITY AND AWARENESS IN MANAGING PACING, SLEEP AND NUTRITION

3F - PREPARE YOURSELF METICULOUSLY FOR THE SCENARIOS YOU WILL FACE

4D - GIVE UP ON MANAGING THE PROBLEM

4F - HAVE PROVEN PROCEDURES TO DEAL WITH THE MOST FREQUENT DIFFICULTIES

5F - EFFECTIVE EMOTIONAL MANAGEMENT OF UNEXPECTED EVENTS

6F - KNOWING HOW TO ASK FOR HELP

7F - ATTENTION AND METACOGNITION

8F - EXPERT MANAGEMENT OF INNER STATES

9F - SET SHORT-TERM GOALS TO DIVIDE THE ENTIRE JOURNEY

Expert B- Expert C

		Expert C												
		1D	1F	2D	2F	3F	4D	4F	5F	6F	7F	8F	9F	Total
Expert B	1D	1	0	0	0	0	0	0	0	1	0	0	0	2
	1F	0	0	0	0	0	0	0	0	0	0	0	0	0
	2D	0	0	0	0	0	1	0	0	0	0	0	0	1
	2F	0	0	0	9	1	0	0	0	0	0	0	0	10
	3F	0	0	0	0	2	0	0	0	0	0	0	0	2
	4D	0	0	0	1	0	0	0	0	0	0	0	0	1
	4F	0	0	0	0	0	0	3	0	0	0	1	0	4
	5F	0	0	0	1	0	0	0	4	0	0	0	0	5
	6F	0	0	0	0	0	0	0	0	2	0	0	0	2
	7F	0	1	0	0	0	0	0	0	0	1	0	0	2
	8F	0	0	0	0	0	0	0	0	0	0	0	0	0
	9F	0	0	0	0	0	0	0	0	0	0	0	1	1
Total		1	1	0	11	3	1	3	4	3	1	1	1	30

Categories:

1D - EXPECTING THE SOLUTION TO COME FROM OUTSIDE

1F - DEVELOP CORRECT EXPECTATIONS

2D - CREATING INCORRECT EXPECTATIONS

2F - FLEXIBILITY AND AWARENESS IN MANAGING PACING, SLEEP AND NUTRITION

3F - PREPARE YOURSELF METICULOUSLY FOR THE SCENARIOS YOU WILL FACE

4D - GIVE UP ON MANAGING THE PROBLEM

4F - HAVE PROVEN PROCEDURES TO DEAL WITH THE MOST FREQUENT DIFFICULTIES

5F - EFFECTIVE EMOTIONAL MANAGEMENT OF UNEXPECTED EVENTS

6F - KNOWING HOW TO ASK FOR HELP

7F - ATTENTION AND METACOGNITION

8F - EXPERT MANAGEMENT OF INNER STATES

9F - SET SHORT-TERM GOALS TO DIVIDE THE ENTIRE JOURNEY
